# Investigation of Ti/Al_2_O_3_ + TiO_2_ and Ti + TiO_2_/Al_2_O_3_ + TiO_2_ hybrid coatings as protection of ultra-light Mg–(Li)–Al–RE alloys against corrosion

**DOI:** 10.1038/s41598-022-23452-x

**Published:** 2022-11-12

**Authors:** Marcin Staszuk, Daniel Pakuła, Łukasz Reimann, Małgorzata Musztyfaga-Staszuk, Robert Socha, Tomasz Tański

**Affiliations:** 1 Department of Engineering Materials and Biomaterials, Silesian University of Technology, Konarskiego Street 18A, 44-100 Gliwice, Poland; 2 Materials Research Laboratory, Silesian University of Technology, Konarskiego Street 18A, 44-100 Gliwice, Poland; 3 Welding Department, Silesian University of Technology, Konarskiego Street 18A, 44-100 Gliwice, Poland; 4 Institute of Catalysis and Surface Chemistry PAS, Niezapominajek Street 8, 30-239 Kraków, Poland; 5 Centrum Badań i Rozwoju Technologii dla Przemysłu S.A., Ludwika Waryńskiego 3A, 00-645 Warszawa, Poland

**Keywords:** Energy science and technology, Engineering, Materials science, Nanoscience and technology

## Abstract

Low corrosion resistance is a significant problem of magnesium alloys, particularly ultra-light magnesium-lithium alloys. Surface treatment is one way to improve their corrosion resistance. The paper presents the results of tests of Ti/Al_2_O_3_ + TiO_2_ and Ti + TiO_2_/Al_2_O_3_ + TiO_2_ coatings obtained in a hybrid process combining PVD and ALD methods and ALD coating of Al_2_O_3_ + TiO_2_ type obtained on AE42 (Mg–4Li–2RE) and LAE442 (Mg–4Li–4Al–2RE). Structural studies were performed using scanning and transmission electron microscopy (SEM and TEM), atomic force microscopy and EDS and XPS spectroscopic methods. Potentiodynamic tests and electrochemical impedance spectroscopy EIS in 0.05 M NaCl solution were performed to determine the electrochemical properties of the tested materials. Moreover, tests of surface wettability and tribological properties using the ball-on-disc method were performed. Based on the analysis of anodic polarisation curves and Tafel analysis, it was found that the Ti + TiO_2_/Al_2_O_3_ + TiO_2_ coating showed the best potentiodynamic properties on both substrates. In particular, on the magnesium-lithium substrate, the value of the polarisation resistance of this hybrid coating is R_pol_ = 14 × 10^3^ Ω × cm^2^, and the value of the corrosion current is j_corr_ = 0.4 µA/cm^2^. For the uncoated LAE442 substrate, the polarisation resistance is R_pol_ = 1.05 × 10^3^ Ω × cm^2^, and the corrosion current value is j_corr_ = 5.49 µA/cm^2^. This improvement is due to the synergistic effect of the combined PVD and ALD technologies. The study confirmed the impact of hybrid coatings on improving the anti-corrosion and tribological properties of ultra-light magnesium alloys.

## Introduction

For many years, due to excellent properties such as high specific strength, high damping capacity, and the required recyclability and low density, magnesium and lithium alloys have been widely used primarily in commonly used consumer electronics and the automotive industry. The addition of lithium in the magnesium alloys results in better formability and a very low density. Conversely, adding aluminium to Mg–Li alloys improves the strength and increases the density slightly, reducing elongation. Apart from these undoubted advantages, the Mg–Li alloy has the following disadvantages: low hardness and poor corrosion resistance. While the mechanical properties of magnesium alloys can be effectively improved by modifying the chemical composition and heat and plastic treatment, a big problem is the improvement of their corrosion resistance^1–5^.

For this purpose, these materials' surface treatment is increasingly often used, looking for optimal coating materials in a single or multiphase system. The big problem is to find such a coating or layer system that is "multifunctional", i.e., resistant not only to corrosion but also chemically stable, non-toxic, has good optical and electrical properties, excellent hydrophilic and hydrophobic properties, and good photocatalytic properties after exposure to UV light. The analysis of research works shows that such a wide range of physicochemical properties can be ensured, in particular, by metal oxides obtained by physical vapour deposition (PVD) and chemical vapour deposition (CVD) methods, including the technique of atomic deposition of ALD layers^6–23^.

Among the coatings obtained by the PVD method on the substrate of AZ91D magnesium alloys with excellent physicochemical properties, high corrosion resistance, hardness and abrasion resistance, the ZrO_2_ coating applied with the RFPVD technique can be distinguished, which was confirmed by the authors in^10^. Another PVD coating that, in particular, improves corrosion resistance is the ZnO coating and the ZnO/MWCNT duplex coating. In the research work^11^, it was found that the chemically inert MWCNT layer obtained by immersion coating filled the imperfections (micropores and microcracks) of the ZnO coating obtained by the PVD technique, preventing the formation of corrosion centres in the coating, thereby increasing the corrosion resistance, in this case, it was the alloy substrate magnesium Mg–0.8Ca–3Zn. Using the PVD magnetron sputtering method, as part of the research in^12^, various coatings were applied to the substrate of the Mg–3Sn alloy: a Si_1−*x*_C_*x*_ layer, a Si_1−*x*_C_*x*_ coating with an Mg intermediate layer and a combination of Mg/AlTi/Si_1−*x*_C_*x*_ layers. The authors showed that the Si_1−*x*_C_*x*_ coating improved the thermal conductivity of the material and its electrical resistivity and corrosion resistance without reducing the properties of the magnesium alloy itself. In turn, the AlTi interlayer improved the adhesion of the coating to the base material^12^.

One of the promising methods of producing this type of coating materials, in addition to PVD techniques, is the method of atomic deposition of ALD layers, which allows for obtaining layers of high quality and controlled thickness, even on surfaces with complex shapes. With this method, we can obtain many oxides, TiO_2_, ZrO_2_, SiO_2_, CeO_2_, and Al_2_O_3_, which primarily provide high corrosion resistance and abrasion resistance, high chemical and thermal stability, electrical stability and heat insulation, high hardness and strength. In ^13^, the authors showed an improvement in the corrosion resistance of AZ31 magnesium alloy with a 100 nm thick ZrO_2_ coating. Many studies show that the TiO_2_ phase, in particular with an amorphous structure, has excellent anti-corrosion properties as a single layer (applied on a magnesium alloy—work^14^) or as a sealing layer in a PVD/ALD hybrid coating PVD coating (obtained on substrates of resistant steel on corrosion and aluminium alloys ^15,16^). The authors confirmed the advantages of PVD/ALD hybrid coatings on various substrates by testing the following coating systems: CrN/Al_2_O_3_ + TiO_2_, TiAlN + TiN/Al_2_O_3_, TiCN/Al_2_O_3_ and the bimodal TiO_2_/nanoTiO_2_ coating. The multilayer coating systems discussed lead to an increase in corrosion resistance and a decrease in the corrosion current density of the coating. Relative to the substrate, the corrosion resistance of such coatings increases by several tens of per cent. In addition, the applied nitride phases obtained by PVD techniques in these coating systems raise the hardness of the entire system, thereby contributing to an increase in abrasion resistance. In some cases, improved hydrophobicity has also been observed^15–22^.

The above literature analysis shows that multilayer hybrid coatings provide better material corrosion resistance than single layers because the outer layer fills the discontinuities in the intermediate layer, sealing it and eliminating the possibility of the corrosive agent penetrating the coating into the substrate through defects in the intermediate layer. Increased barrier properties of penetration of the corrosive agent for ALD coatings in multilayer systems have also been noticed. Usually, the Al_2_O_3_ layer as an excellent permeation barrier is combined with another chemically more durable layer, e.g. Al_2_O_3_ + SiO_2_, Al_2_O_3_ + TiO_2_, Al_2_O_3_ + HfO_2_ and Al_2_O_3_ + ZrO_2_ nanolaminates. Apart from combining good barrier properties with their chemical durability, another suggested reason for improving the functional properties of the tested coating systems is the elimination of defects in the coating by separating the crystallised layers from the amorphous ones^23^.

As a result of literature analysis and based on their research^14–16^, the authors in the present work applied the following coating systems to magnesium alloy substrates, which have not yet been investigated:

- Ti/Al_2_O_3_ + TiO_2_ and Ti + TiO_2_/Al_2_O_3_ + TiO_2_ were obtained by a hybrid PVD/ALD method.
- Al_2_O_3_/TiO_2_ coating obtained by a single ALD technique.

In these hybrid systems, the Ti (PVD) layer has the task of improving the adhesion of the coating to the substrate. In turn, the PVD TiO_2_ layer should ensure adequate durability and corrosion resistance of the tested materials. On the other hand, the ALD layers, including Al_2_O_3_, an excellent permeability barrier, and the TiO_2_, chemically more durable and has high corrosion resistance, ensure proper sealing of PVD coatings in the hybrid system. In addition, the single-technique ALD coating was tested for comparison. The novelty of this work is that on this type of substrates, such coating systems consisting of these phases (layers) have not been applied and studied in such detail. Therefore, this study investigates the structure and functional properties of Ti/Al_2_O_3_ + TiO_2_ and Ti + TiO_2_/Al_2_O_3_ + TiO_2_ coatings obtained by the hybrid PVD/ALD method and the Al_2_O_3_/TiO_2_ coating obtained by a single ALD technique on magnesium alloy substrates.

## Materials

The coatings were made on magnesium alloys substrates of AE42 type (Mg–4Al–2RE) and LAE442 (Mg–4Li–4Al–2RE) types. Round substrate samples with a diameter of 14 mm and a thickness of about 5 mm were used. Before coating, the substrates were ground and finally polished on a 1 µm abrasive. Before coating, the samples were rinsed in an ultrasonic bath with acetone and dried with compressed air.

Coatings such as Ti/Al_2_O_3_ + TiO_2_ and Ti + TiO_2_/Al_2_O_3_ + TiO_2_ were obtained using the PVD/ALD hybrid method. An Al_2_O_3_/TiO_2_ coating was also deposited with a single ALD technique. The Ti and TiO_2_ layers were deposited by MS-PVD magnetron sputtering using a Kurt J Lesker PVD 75 device (Clairton, PA, USA). Deposition of the layers with the MS-PVD method was preceded by heating the substrates to 100 °C and ionising argon cleaning. The process parameters are presented in Table 1.

**Table 1:** Conditions of deposition of MS-PVD layers.

Parameter, unit	Matter/values
Metal vapour source, target	Ti (purity: 99.995%)
Inert gas	Ar (purity: 99.9999%)
Reactive gas (in the case of a TiO_2_ layer)	O_2_ (purity: 99.9999%)
Temperature proc., °C	100
Deposition time of Ti layer, min	2
Deposition time of TiO_2_ layer, min	120
Power on magnetron source, W	200
BIAS, V	140

A Beneq P400 reactor (Espoo, Finland) was used to deposit the ALD Al_2_O_3_ + TiO_2_ bilayers. Calibration runs for Al_2_O_3_ and TiO_2_ and Al_2_O_3_ + TiO_2_—bilayer were done before the sample run. Thickness and reflective index were measured from silicon monitor pieces from the runs using ellipsometry. The Design of Experiments includes one sample run in total, as shown in Table 2.

**Table 2:** Conditions of deposition of ALD Al_2_O_3_ + TiO_2_ layers.

Parameter, unit	Matter/values
Titanium source	TiCl_4_ (purity: 99.9999%)^a^
Aluminium source	TMA (purity min. 99.999%)^a^
Oxygen source	H_2_O
Temperature, °C	120
Thickness Al_2_O_3_/TiO_2_, nm	20/40^b^

^a^Precursor of Strem Chemicals, Inc.

^b^Thickness was determined by spectroscopic ellipsometry on reference coatings applied to silicon wafer substrates.

## Methodology

Observations of the tested materials' structure, corrosion mechanisms, and tribological wear mechanisms were made using a scanning electron microscope (SEM) Zeiss Supra 35 (Zeiss, Oberkochen, Germany). The research was carried out using the detection of backscattered electrons (SE and InLens detectors), and the chemical composition in the micro-regions was analysed using characteristic X-ray energy (EDS) detection. The tests were performed at an accelerating voltage range of 5–20 kV.

Morphology studies were performed using the Park System XE-100 atomic force microscope (AFM) (Suwon, Korea) in a non-contact mode. A non-contact mode was used with a probe elastic constant of 40 N/m and a resonance frequency of 300 kHz.

The tests of thin films from the cross-section of hybrid coatings were carried out using the Titan 80–300 high-resolution scanning and transmission electron microscope (S/TEM) by FEI (Eindhoven, The Netherlands). EDS spectroscopy was used to analyse the chemical composition in micro-regions. The tests were performed at an accelerating voltage of 300 kV.

The surface chemical composition was analysed by the XPS method using a photoelectron spectrometer (ESCA/XPS) with an EA15 semispherical analyser (PREVAC). The source of Mg Kα radiation (1253.6 eV) was the RS 40B1 lamp (PREVAC) with a power of 180 W. The energy resolution of the spectrometer for the Ag 3*d*_5/2_ line was 0.9 eV (for the analyser transition energy equal to 100 eV). The spectrometer has been calibrated per ISO 15472: 2010. The vacuum level during the spectra measurement was about 1 × 10^–9^ mbar.

The studies of electrochemical properties were performed with the potentiodynamic method and electrochemical impedance spectroscopy EIS in 0.05 M NaCl solution using the potentiostat/galvanostat ATLAS 0531EU by Atlas Sollich (Rębiechowo, Poland). Corrosion tests were performed in a three-electrode system: the reference electrode was an Ag/AgCl electrode, and the auxiliary electrode was made of stainless steel wire. The corrosion resistance tests were carried out in two stages:

1. Determination of the open circuit potential (E_ocp_) for 1 h;
2. The potentiodynamic method from starting potential E_start_ = E_ocp_–100 mV to E_finish_ = 1 V or current density 1 mA/cm^2^, the rate of potential increase was 1 mV/s.

Characteristic electrical quantities describing corrosion resistance, i.e. current density (j_corr_) and corrosion potential (E_corr_), as well as polarisation resistance (R_pol_), were determined using the Tafel method and AtlasLab software.

The second research method was impedance spectroscopy (EIS), in which, first, for 15 min, the samples were stabilised in the test environment without current flow and then with forced flow through a solidified AC system at an amplitude of 10 mV in the frequency range from 100 kHz to 10 MHz. The results are presented in the form of Nyquist and Bode charts. To accurately reproduce the relations appearing in the examined electrochemical process, they were assigned a substitute electrical system, using the AtlasLab and EC-Lab software, in which the numerically generated curves were fitted to those recorded in the experiment, and apart from typical resistors and an inductor, fixed-phase CPE elements (CPE) were used (Constant Phase Element).

The tribological properties of the coatings were determined by the ball-on-plate method using a CSM Instruments Standard Tribometer. The counter sample was a WC–Co cemented carbide ball with a diameter of 6 mm. The test conditions were as follows: room temperature, humidity approx. 50%, linear speed v = 0.5 cm/s, normal force Fn = 0.5 N, diameter of the wipe path 5 mm. The maximum number of cycles is 500, with one cycle constituting one complete revolution of the test sample. The friction force was recorded during the test, and the friction coefficient µ was determined.

## Results and discussion

**Figure 1:**
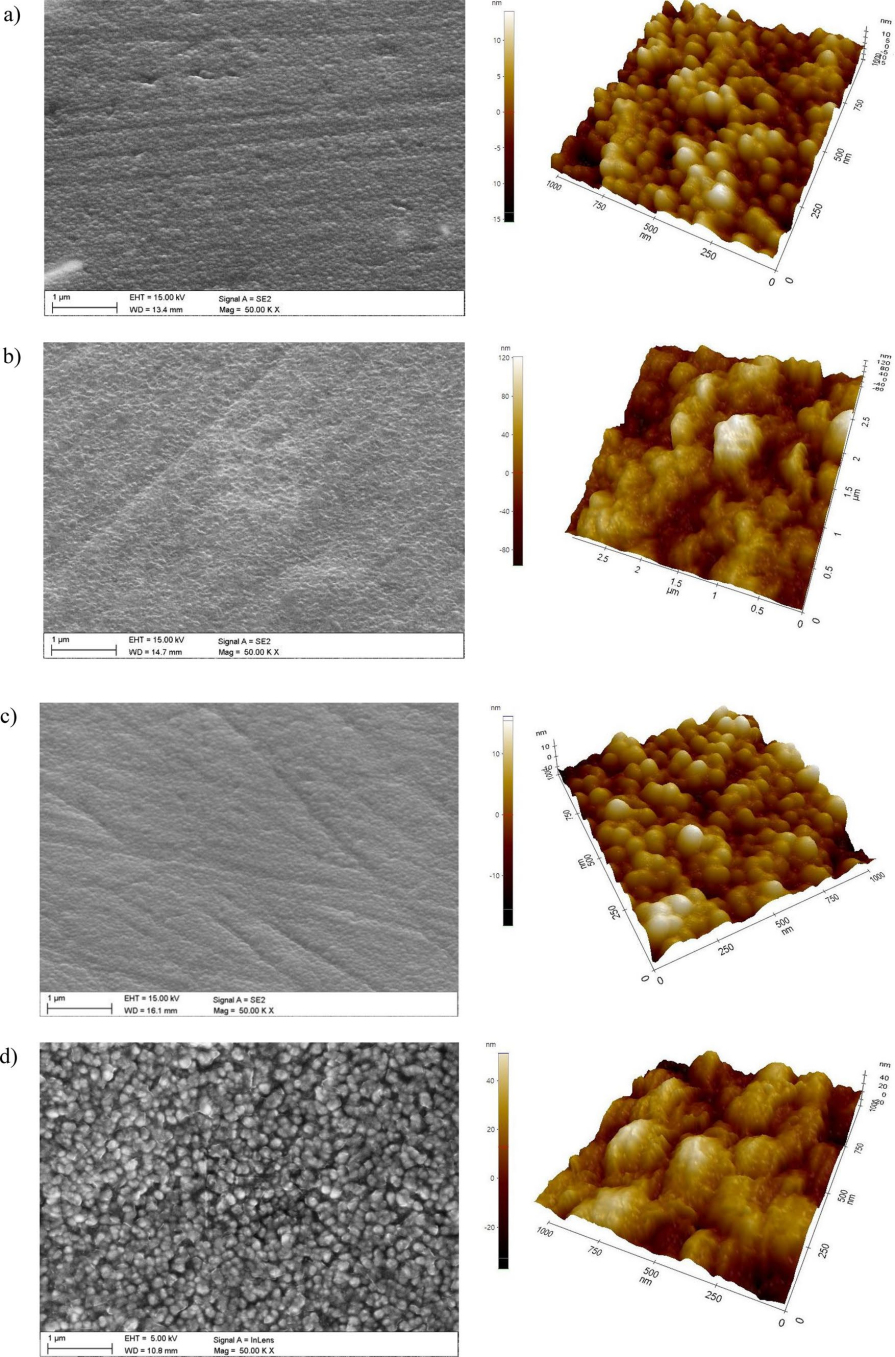
Morphology of PVD/ALD coatings (SEM, AFM): (**a**) Ti/Al_2_O_3_ + TiO_2_ on AE42, (**b**) Ti/Al_2_O_3_ + TiO_2_ on LAE442, (**c**) Ti + TiO_2_/Al_2_O_3_ + TiO_2_ on AE42, (**d**) Ti + TiO_2_/Al_2_O_3_ + TiO_2_ on LAE442.

**Figure 2:**
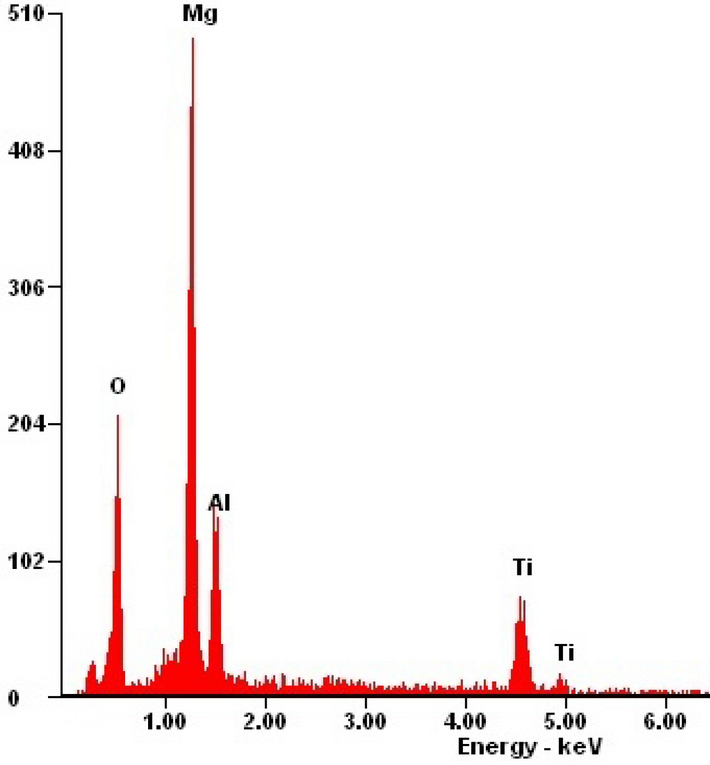
X-ray energy-dispersive plot of the area shown in Fig. 1.

As a result of the research on the morphology of hybrid coatings, it was found that the coatings have a compact structure without pores and discontinuities (Fig. 1). There are unevennesses (scratches) on the surface of the tested coatings, which are the residues of substrate preparation for coating by grinding and polishing. Based on the analysis of the results of morphology studies in the AFM atomic force microscope, it was found that the type of the covered substrate significantly affects the morphology. Both tested hybrid coatings on the substrate made of magnesium alloy without lithium AE42 (Fig. 1a,c) show a granular structure on the nanoscale with an average grain size of about 80 nm. In the case of substrates made of magnesium alloys with LAE442 lithium, the morphology of the tested coatings is composed of clusters with a size of 300–800 nm consisting of sub-grains with a size in the range from 160 to 260 nm (Fig. 1b,d). Analysis of the chemical composition using the EDS method on the tested surfaces confirmed the presence of titanium and aluminium elements suitable for hybrid coatings. In addition, there is an intense reflection from the magnesium substrate (Fig. 2).

**Figure 3:**
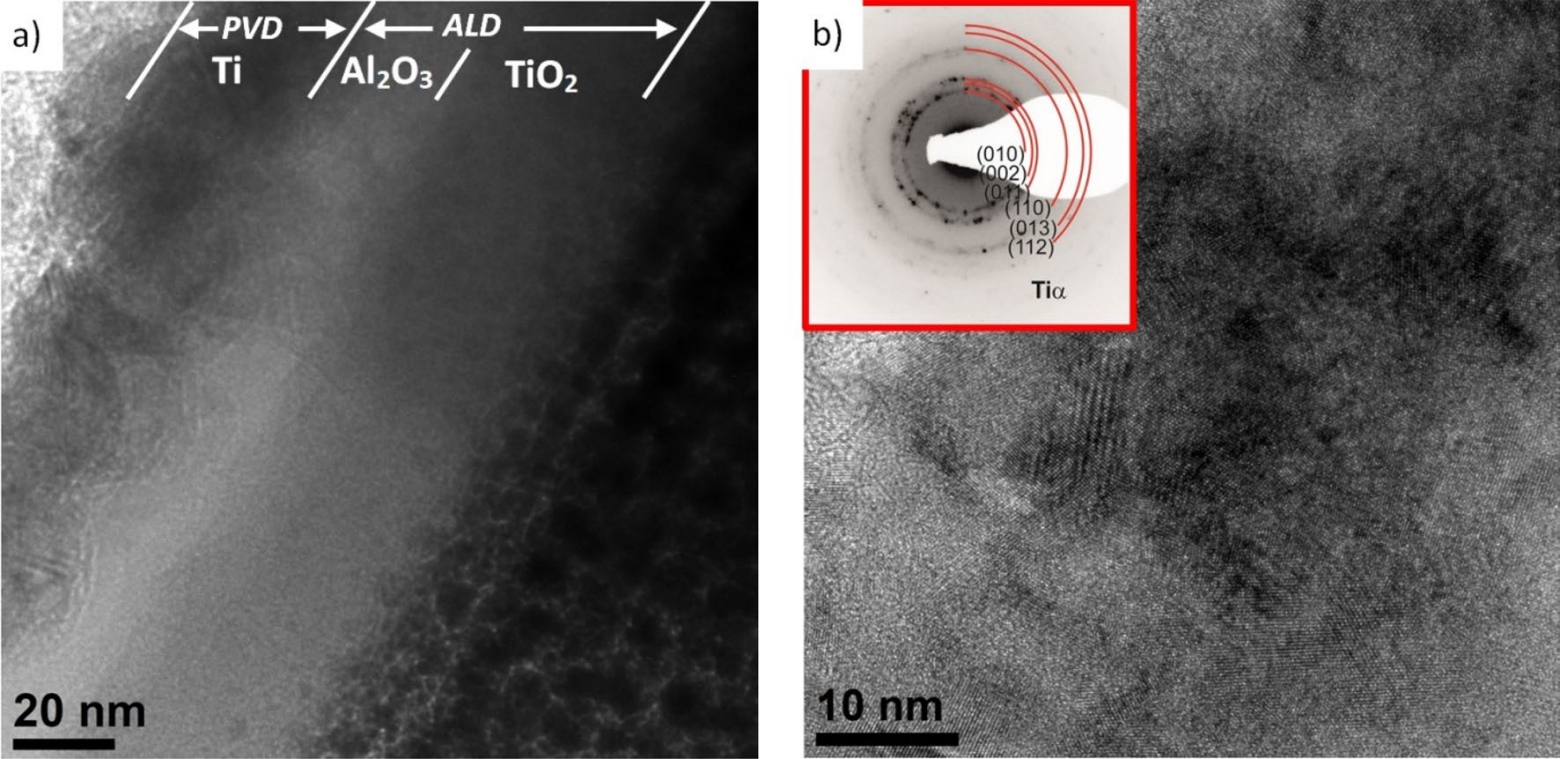
Structure (HR-TEM) of Ti/Al_2_O_3_ + TiO_2_ hybrid coating on AE 42 alloy substrate: (**a**) the entire cross-section of the coating, (**b**) Ti layer (with diffraction pattern).

**Figure 4:**
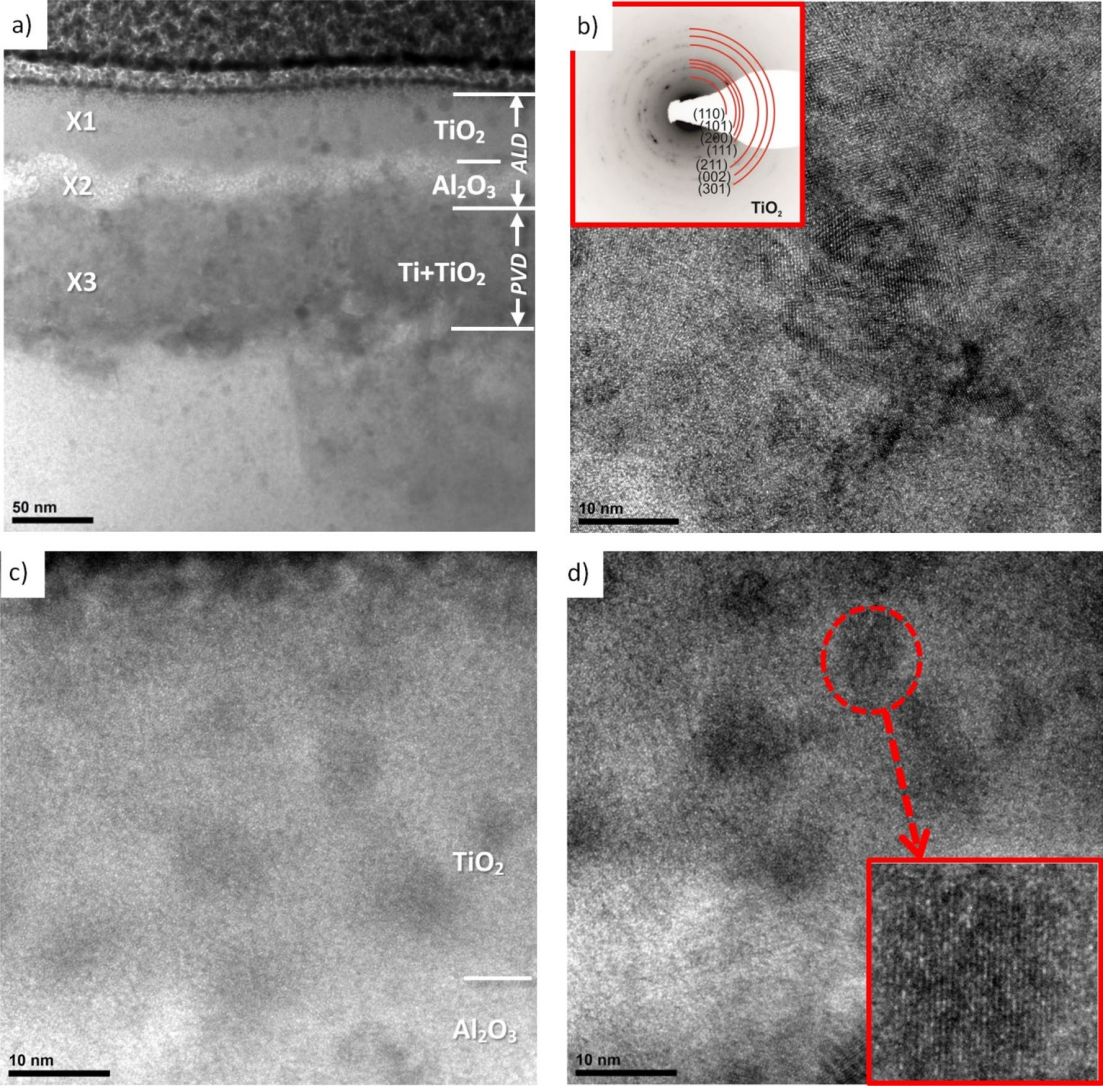
Structure (HR-TEM) of Ti + TiO_2_/Al_2_O_3_ + TiO_2_ hybrid coating on AE 42 alloy substrate: (**a**) the entire cross-section of the coating, (**b**) TiO_2_ PVD layer (with diffraction pattern) (**c**,**d**) Al_2_O_3_ + TiO_2_ ALD layers.

Examination of the structure of thin films from the cross-section of both hybrid coatings on AE 42 alloy substrate in a high-resolution transmission electron microscope confirmed the layered structure of the coatings (Figs. 3, 4). The thickness of the PVD layers is Ti = 31 nm and TiO_2_ = 92 nm in the Ti/Al_2_O_3_ + TiO_2_ and Ti + TiO_2_/Al_2_O_3_ + TiO_2_ coatings, respectively. The thicknesses of the ALD layers are respectively Al_2_O_3_ = 20 nm and TiO_2_ = 40 nm and are consistent with the assumptions and ellipsometric measurements of the thickness of the control layers on silicon substrates. Both layers obtained by the PVD method show crystal structures. Structural analysis using electron diffraction showed that the titanium layer in the Ti/Al_2_O_3_ + TiO_2_ coating has a hexagonal lattice (P63/mmc) Ti-α, while the titanium oxide in the Ti + TiO_2_/Al_2_O_3_ + TiO_2_ coating has a tetragonal lattice (P42mnm) TiO_2_-rutile.

Moreover, based on the presented research, it is not easy to distinguish the phase boundary between the titanium layer and titanium oxide in the Ti + TiO_2_ bilayer obtained by the PVD technique. Observations of the structure of the ALD layers in the bright field with high resolution allowed us to establish that they show the presence of single cubic crystals in the amorphous matrix (Fig. 4c,d). The crystallite sizes range from 4 to 8 nm. The structure obtained results from the nucleation of crystalline phases in an amorphous matrix.

**Figure 5:**
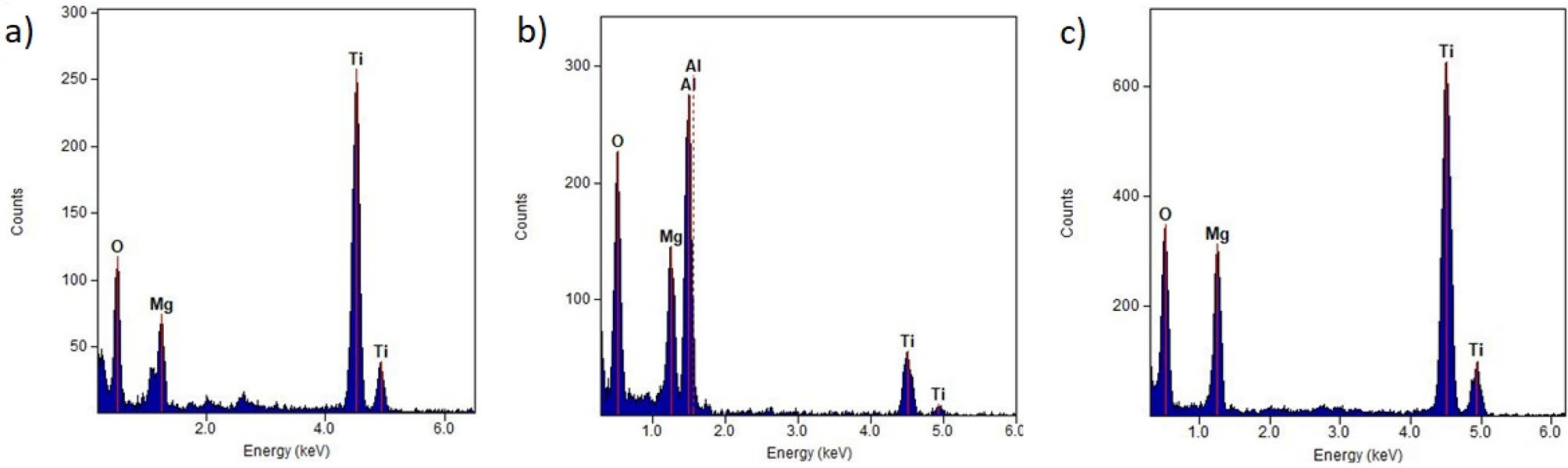
X-ray energy-dispersive plot as Fig. 4a of the area: (**a**) X1, (**b**) X2, (**c**) X3.

Moreover, the chemical composition analysis in the micro-areas confirmed the presence of chemical elements suitable for a given layer (Fig. 5). The spectrograms from the areas of titanium oxide layers (Fig. 5a,c) (micro-areas X1 and X3 according to (Fig. 4a) are dominated by reflections from titanium. On the other hand, in the EDS spectrum from the aluminium oxide micro-area (Fig. 5b) (micro-area X2), the reflex from aluminium dominates. All spectra have reflections from oxygen and magnesium. The presence of magnesium in the coating area is undoubtedly a side effect of the preparation of a thin film preparation for testing in a TEM microscope using the FIB ion etching method. On the other hand, oxygen is a component of the tested coatings.

**Figure 6:**
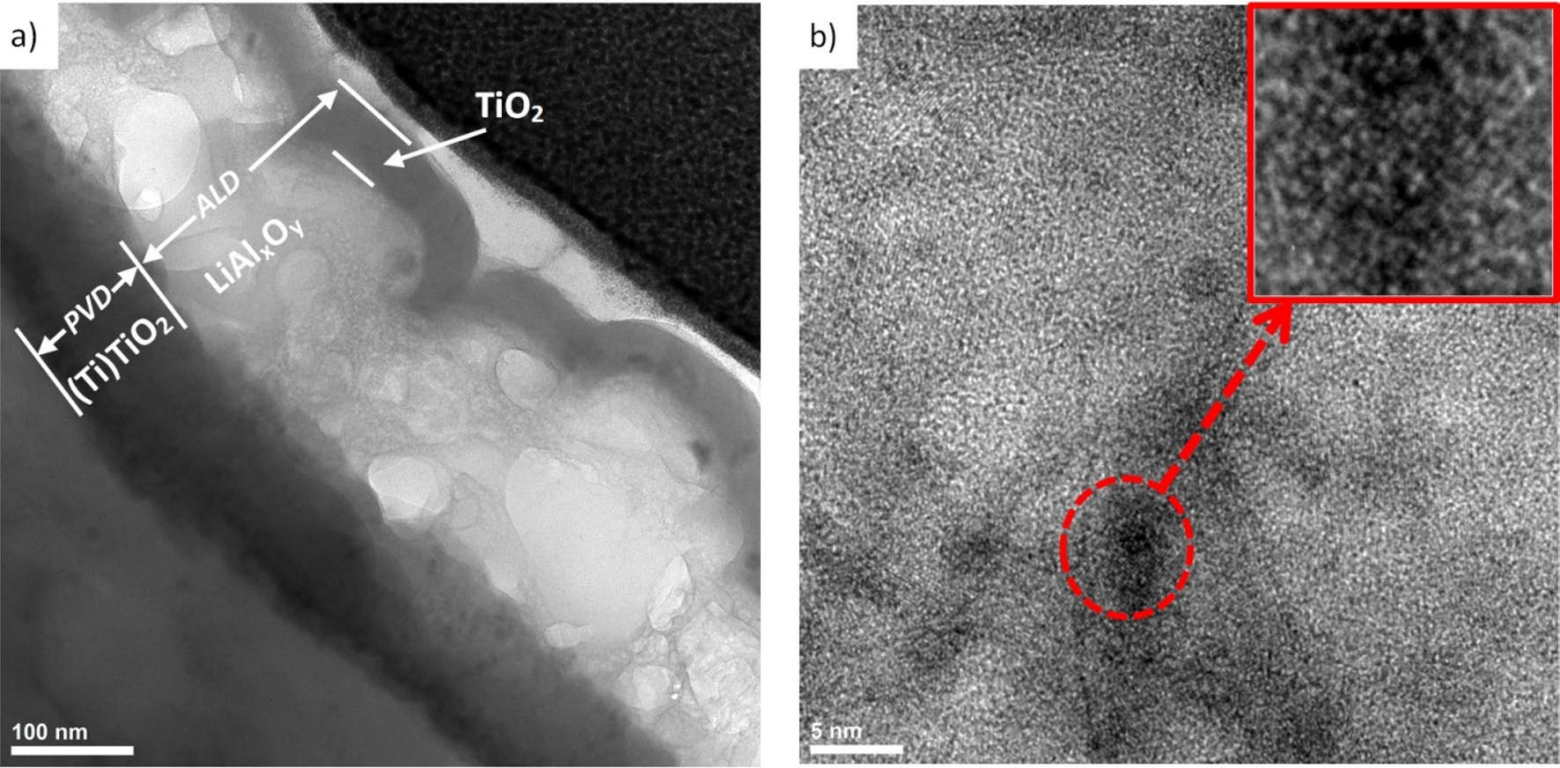
Structure (HR-TEM) of Ti + TiO_2_/Al_2_O_3_ + TiO_2_ hybrid coating on LAE 442 alloy substrate: (**a**) the entire cross-section of the coating, (**b**) Al_2_O_3_ (LiAl_*x*_O_*y*_) ALD layer.

TEM investigations of Ti + TiO_2_/Al_2_O_3_ + TiO_2_ hybrid coating on magnesium-lithium alloy substrate of LAE 442 type showed different structural structures compared with this coating on a lithium-free substrate (Fig. 6). This coating also shows a layered structure. However, the aluminium oxide layer is thicker, i.e., the average thickness is 200 nm. The thicknesses of the other layers are similar to those of the corresponding coating on the lithium-free substrate. Moreover, the Al_2_O_3_ layer is amorphous with MRO (Medium Range Order) regions of atoms. The layer resembles sea foam in its structure, which is related to the formation of LiAlxOy solution due to lithium diffusion from the substrate. Similar results were obtained in works^24,25^. Lithium diffusion from the substrate is possible through the titanium oxide layer obtained by the PVD method because lithium has a high diffusion capacity in rutile^26^.

**Figure 7:**
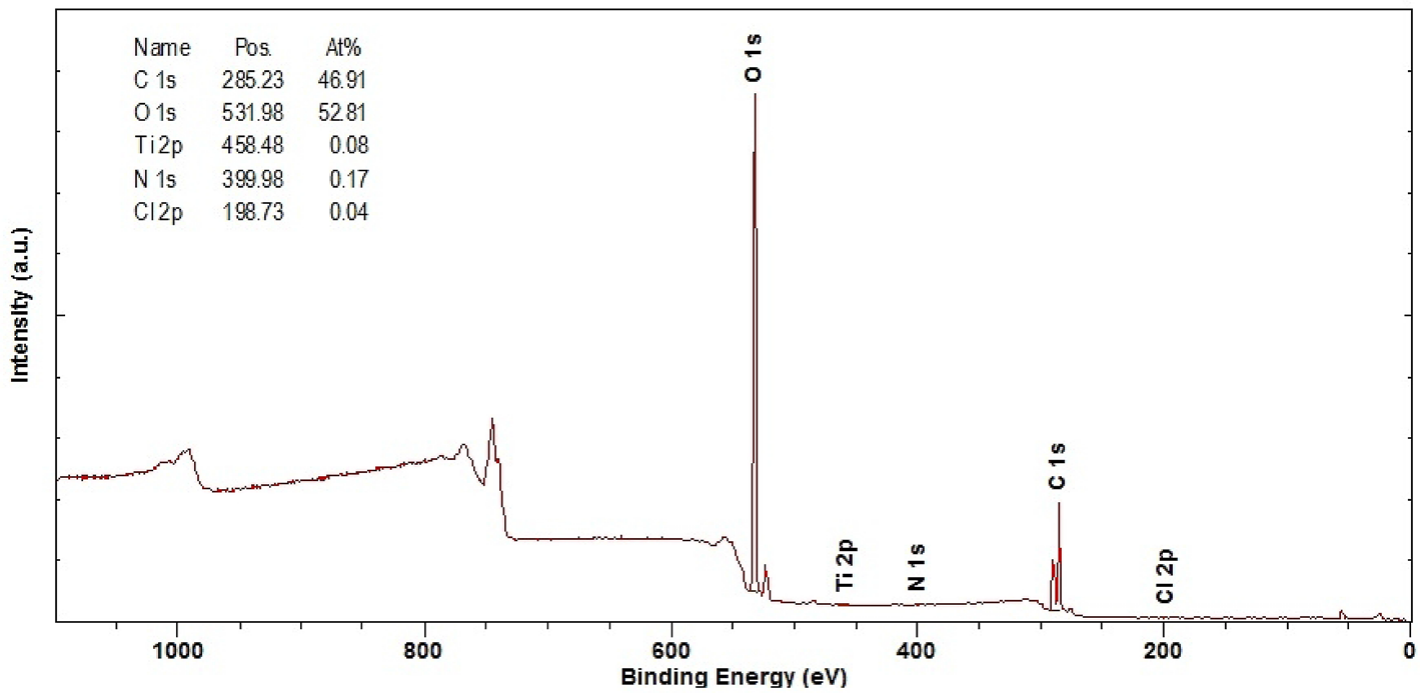
XPS spectrum of Ti + TiO_2_/Al_2_O_3_ + TiO_2_ coating on LAE442 substrate.

**Figure 8:**
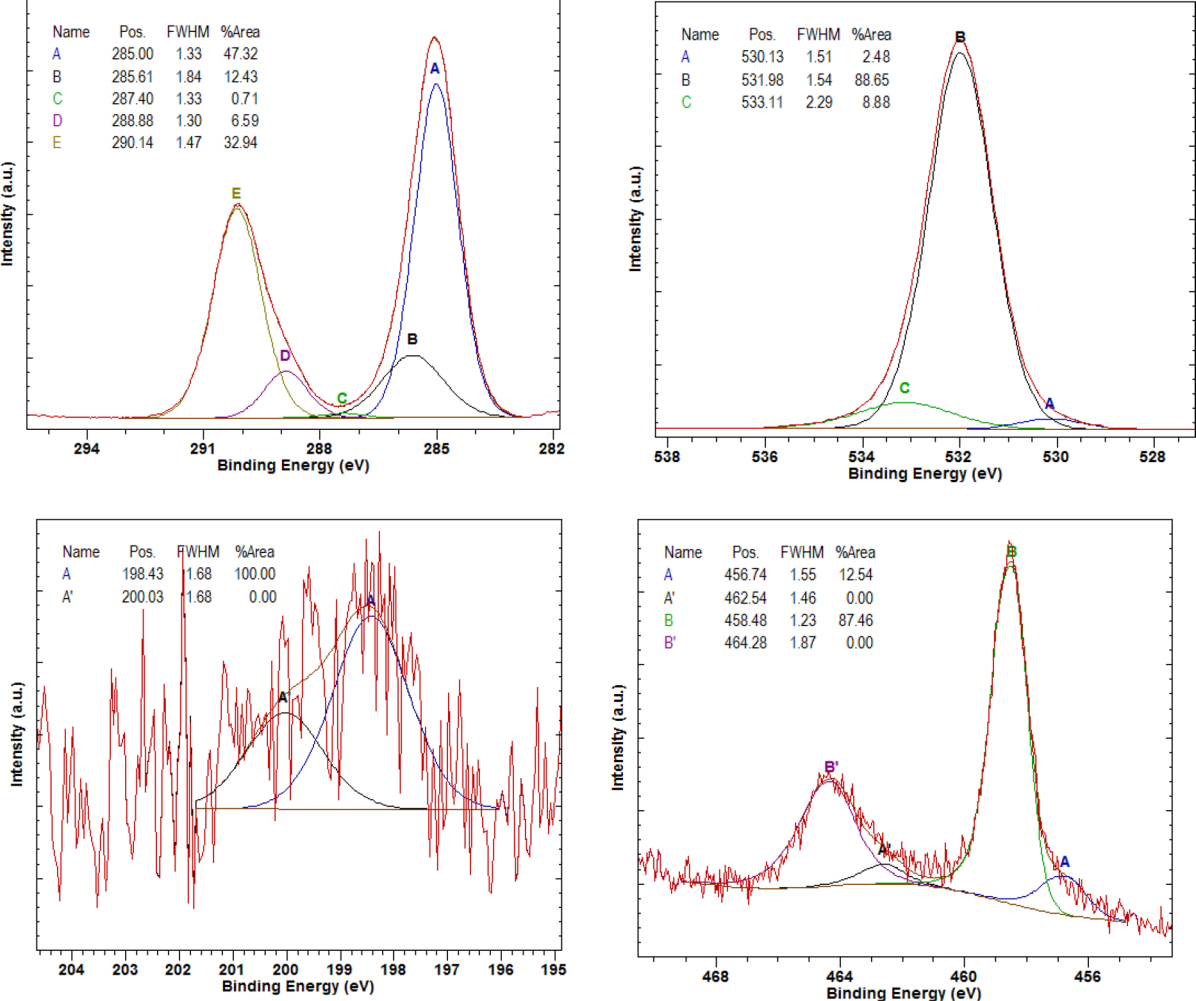
XPS spectra for Ti + TiO_2_/Al_2_O_3_ + TiO_2_ coating on LAE442 substrate: (**a**) C 1*s* spectrum (A: C–C, B: C–OH + C–O–C, C: C = O, D: COOH, E: CO_3_^2−^), (**b**) O 1*s* spectrum (A: O-metal, B: O-C, C: H_2_O), (**c**) Cl 2*p* spectrum (A: Cl-metal), (**d**) Ti 2*p* spectrum (A: Ti^3+^–O, B: Ti^4+^–O).

As a result of the XPS examination of the Ti + TiO_2_/Al_2_O_3_ + TiO_2_ coating deposited on the LAE442 substrate, it was found that the survey spectrum showed photoelectric lines characteristic of the O1*s* and Ti2*p* elements belonging to the surface layer of the tested coating (Fig. 7). Moreover, the presence of N1*s*, C1*s* and Cl2*p* lines was found. Based on the line intensity and the obtained concentrations, it can be concluded that the tested coating is covered with a relatively thick organic oxide layer containing nitrogen and chlorine. Detailed spectra of carbon 1*s*, oxygen 1*s*, chlorine 2*p* and titanium 2*p* were also analysed (Fig. 8). It was found that there are adsorbed impurities on the surface of the tested coating, in particular, organic carbon compounds (short aliphates), water, alcohol and ether groups, carbonyl and carboxyl and carbonate groups. Chlorides were also found. The components of Ti^3+^ –O and Ti^4+^ –O were also found, which correspond to titanium oxides Ti_2_O_3_ and TiO_2_.

**Figure 9:**
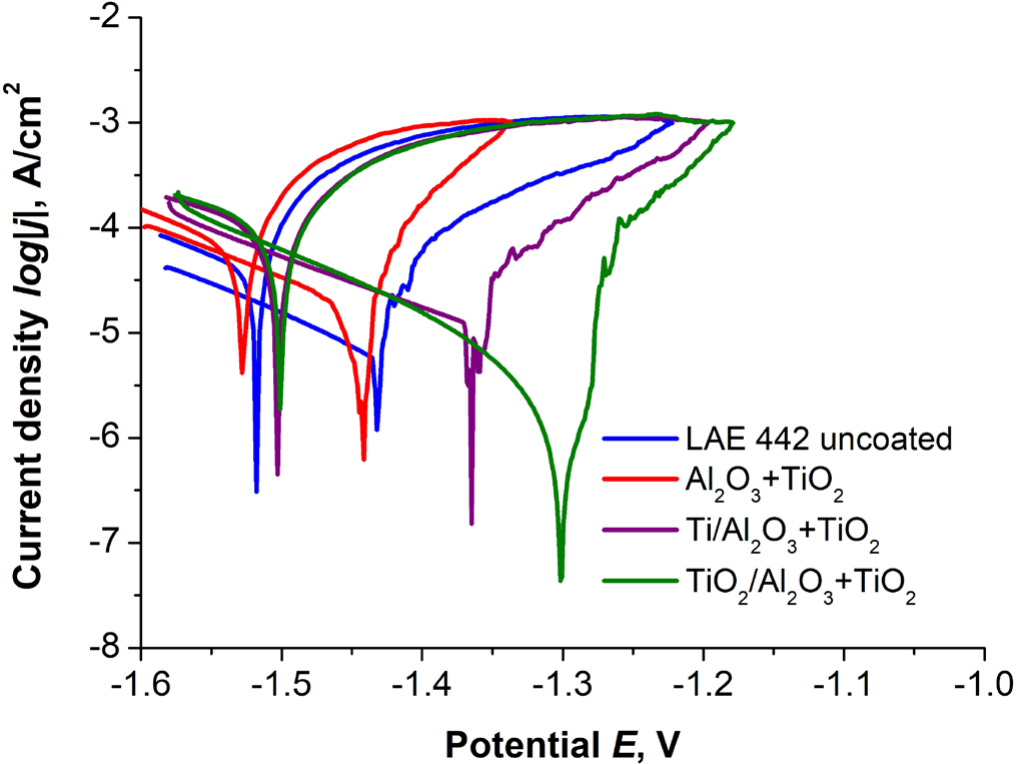
Potentiodynamic polarisation curves for uncoated and coated LAE442 alloy.

The corrosion resistance of the produced materials, depending on the coatings used, was performed in a potentiodynamic test by recording the anodic polarisation curves (Fig. 9), which allowed the use of the Tafel extrapolation method, the results of which are presented in Table 3.

**Table 3:** Potentiodynamic polarisation parameters for samples in 0.05 M NaCl solution.

Substrate	Coating	E_corr_, mV	j_corr_, µA/cm^2^	R_pol_, Ω × cm^2^	P_e_, %^a^
**AE 42**	Uncoated	− 1328	3.80	4250	–
	Al_2_O_3_ + TiO_2_	− 1362	0.56	2659	85
	Ti/Al_2_O_3_ + TiO_2_	− 1356	1.62	1515	57
	Ti + TiO_2_/Al_2_O_3_ + TiO_2_	− 1020	0.33	5400	91
**LAE 442**	Uncoated	− 1430	5.49	1049	–
	Al_2_O_3_ + TiO_2_	− 1441	3.71	1102	32
	Ti/Al_2_O_3_ + TiO_2_	− 1366	2.38	1077	57
	Ti + TiO_2_/Al_2_O_3_ + TiO_2_	− 1298	0.40	14,368	93

^a^P_e_—corrosion protection efficiency$\left(1-\frac{{j}_{corr\,coating}}{{j}_{corr\,substrate}}\right)\times 100\%$.

By analysing the characteristic values determined by the Tafel method, it can be concluded that the coated materials, both in the case of AE42 alloy and LAE442 alloy, were characterised by lower corrosion current density values in relation to the base material. The reduction of the j_corr_ value shows that the tested samples with coatings are characterised by higher resistance to the corrosive effects of the test environment. However, for the AE42 alloy with the Al_2_O_3_ + TiO_2_ coating and the samples with the Ti/Al_2_O_3_ + TiO_2_ coating, the polarisation resistance value decreased from 4.2 kΩ cm^2^ for the uncoated sample up to 2.7 and 1.5 kΩ cm^2^, respectively. At the same time, no significant differences were recorded in the corrosion potential values for these three materials. However, the best improvement in resistance was demonstrated for the sample with Ti + TiO_2_/Al_2_O_3_ + TiO_2_ coating, which was characterised by the lowest corrosion current density of 0.33 µA/cm^2^ among the tested materials, the highest polarisation resistance of 5.4 kΩ cm^2^ and a shift to more positive values by 300 mV with corrosion potential.

Also, for the LAE442 alloy, an improvement in corrosion resistance was observed after applying coatings to its surface, and similarly to the AE42 alloy, the best results were obtained for the Ti + TiO_2_/Al_2_O_3_ + TiO_2_ coating, the current density of which decreased to 0.4 µA/cm^2^ compared to the value of 5.5 µA/cm^2^ for the substrate, and the polarisation resistance increased from 1.0 kΩ cm^2^ to 14 kΩ cm^2^. The corrosion potential for the investigated alloy also shifted to the right, as for the AE42 alloy, due to the substrate being covered with coatings, and the most significant difference of + 150 mV was observed after the deposition of the Ti + TiO_2_/Al_2_O_3_ + TiO_2_ coating. In addition, corrosion protection (Pe), expressed as the ratio of the corrosion current density of the tested coating (j_corr coating_) to the uncoated substrate (j_corr substrate_), shows the highest value for Ti + TiO_2_/Al_2_O_3_ + TiO_2_ coating on both substrates, which is more than 90% in both cases.

**Figure 10:**
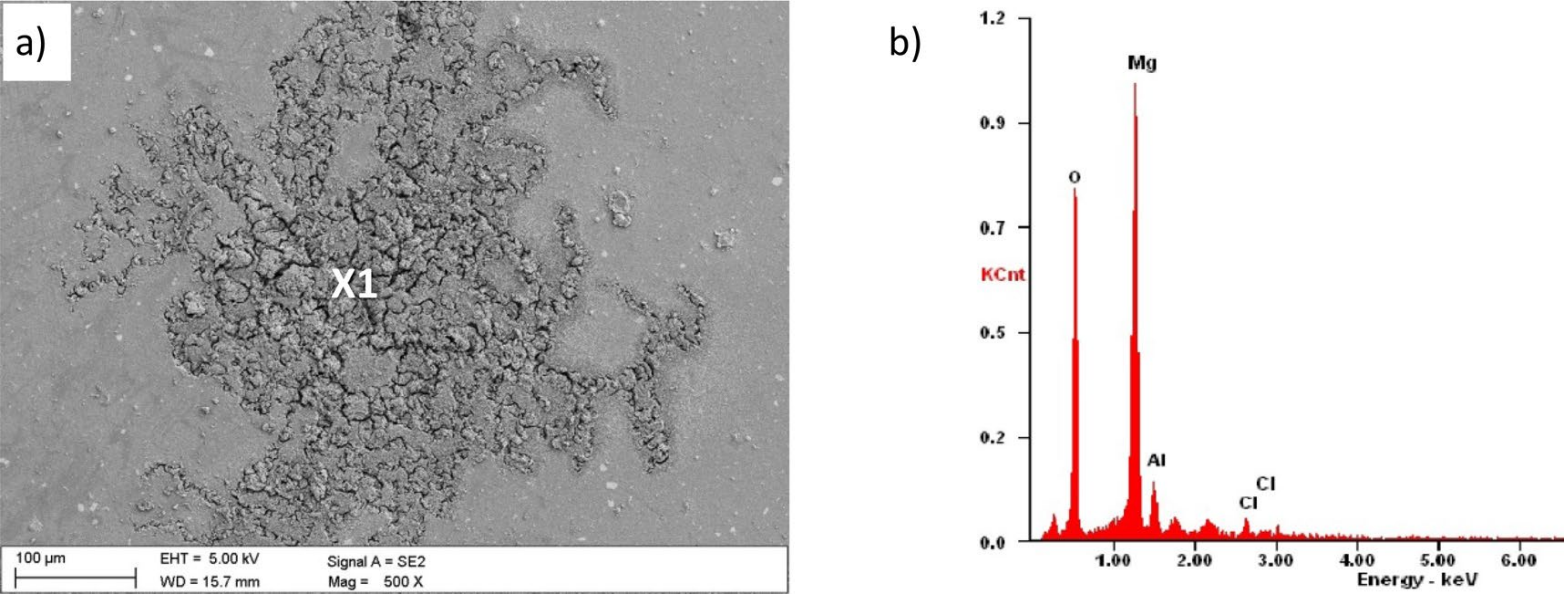
(**a**) Surface morphology after an electrochemical examination of the uncoated LAE442 sample, (**b**) X-ray energy dispersive plot of the area X1 shown in (**a**).

The evaluation of the corrosion mechanisms of the tested materials after potentiodynamic tests was based on the SEM scanning electron microscope (Fig. 10 ÷ Fig. 13). The primary corrosion mechanism is pitting corrosion. Pitting takes various shapes, including round, oblong and irregular shapes. The sizes of corrosion centres in the case of uncoated substrates are 300–700 µm and in the case of surfaces covered with the tested coatings, in the range of 150–500 µm. The observed corrosion damages include cracks and delamination of the coating and slight delamination of the PVD/ALD layers visible at the edges of the cracks of the coatings (Fig. 12c).

**Figure 11:**
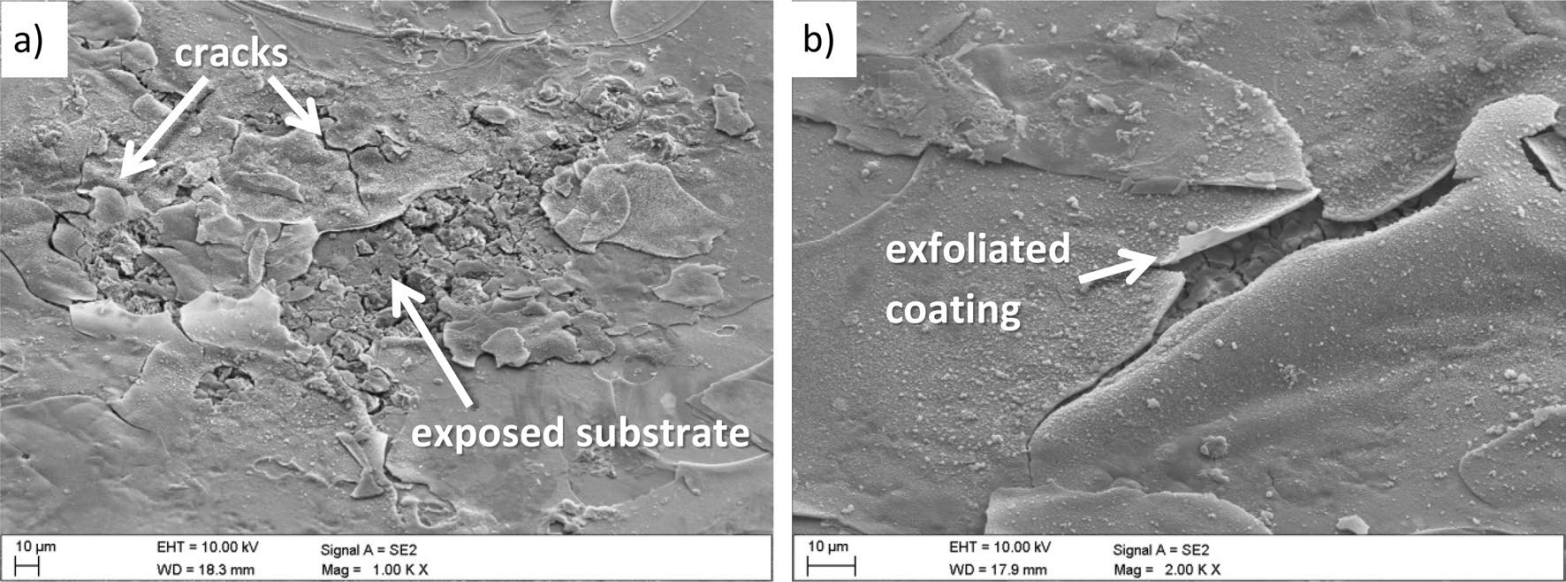
Surface morphology of Al_2_O_3_ + TiO_2_coating on the LAE442 alloy substrate after the corrosion process.

**Figure 12:**
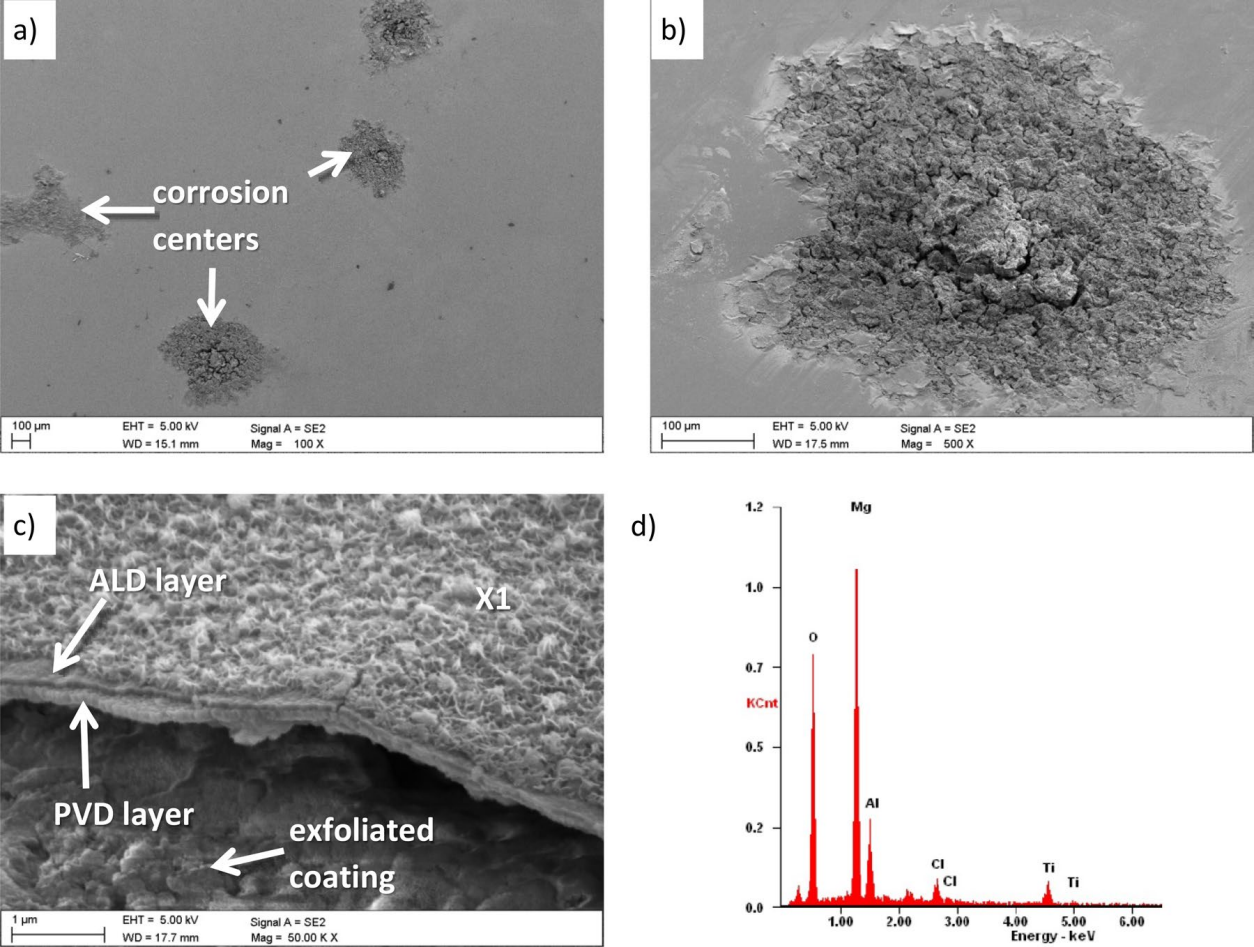
(**a**–**c**) Surface morphology of Ti + TiO_2_/Al_2_O_3_ + TiO_2_ coating on the AE42 alloy substrate after corrosion process, (**d**) X-ray energy dispersive plot of the area X1 shown in (**c**).

**Figure 13:**
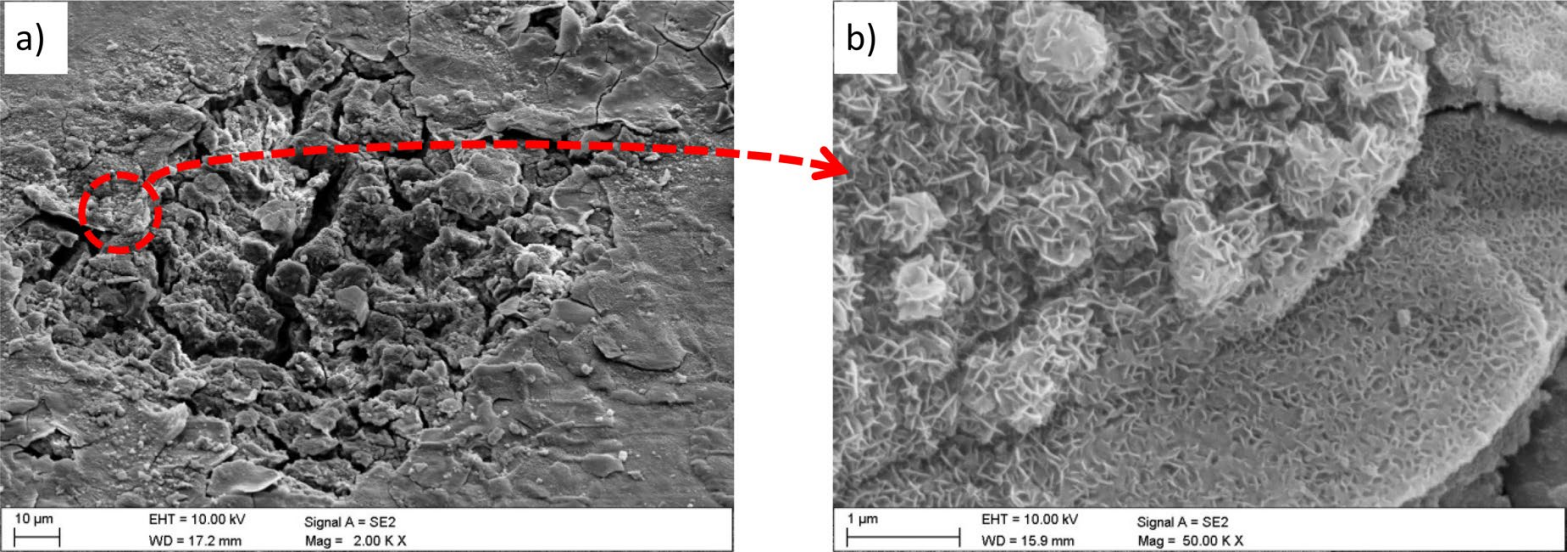
(**a**) Surface morphology of Ti + TiO_2_/Al_2_O_3_ + TiO_2_ coating on the LAE442 alloy substrate after the corrosion process, (**b**) view of crystallised magnesium oxides on the surface of the coating near the corrosion pitting.

Moreover, the chemical composition analysis in the micro-areas of corrosion pitting both uncoated magnesium alloys (Fig. 10b) and exposed substrates of coated samples show the presence of the following elements: Mg, Al, O, and Cl. The presence of these elements indicates the formation of magnesium oxides and magnesium chlorides as products of corrosive processes. Analysis of the chemical composition from the area of the coating near the delamination and pitting showed a similar set of elements with additionally occurring titanium (Fig. 12d). The presence of these elements and microscopic observations (Figs. 11b, 12b,c, 13b) indicate the crystallisation of magnesium oxides and chlorides on the surface of the coating in the area around the cracks and delamination of the coatings.

**Figure 14:**
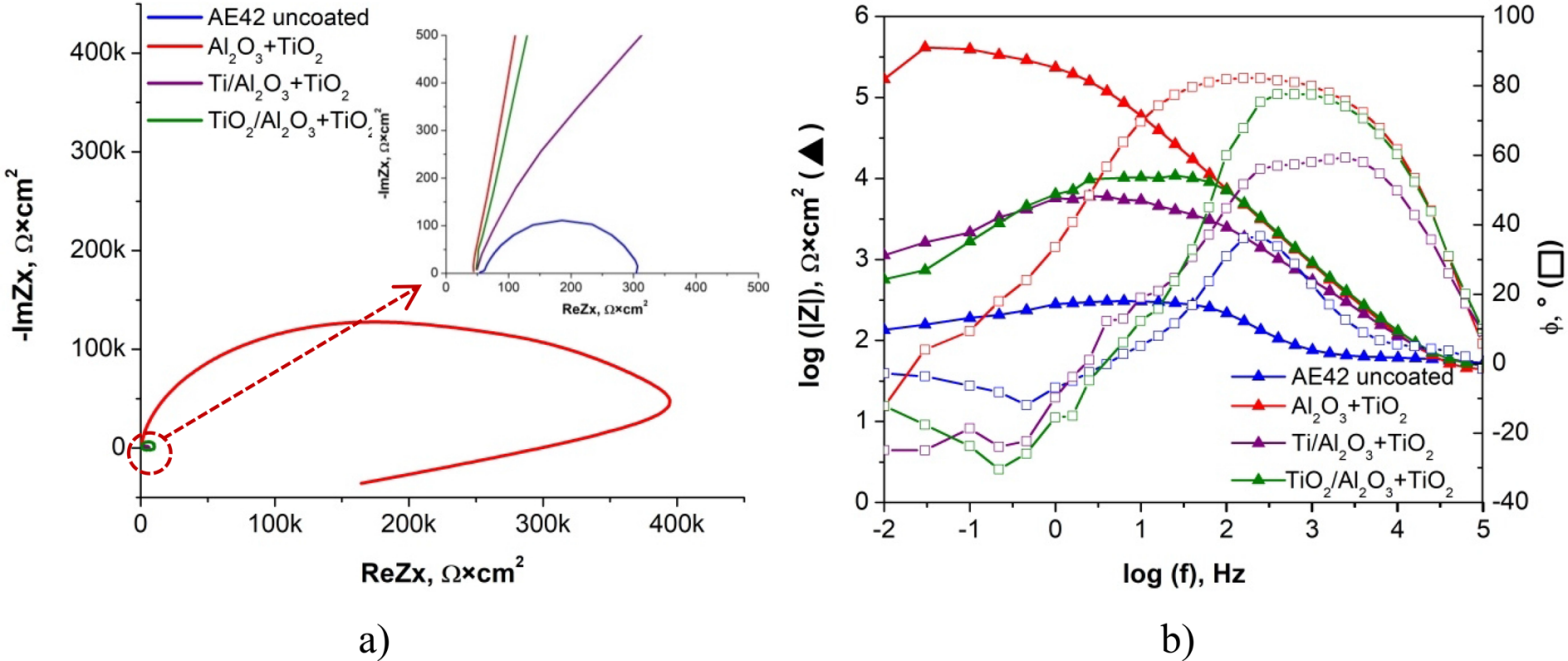
Results of electrochemical impedance spectroscopy (EIS) for uncoated and coated AE42 alloy substrate: (**a**) the Nyquist representation, (**b**) the Bode representation.

**Figure 15:**
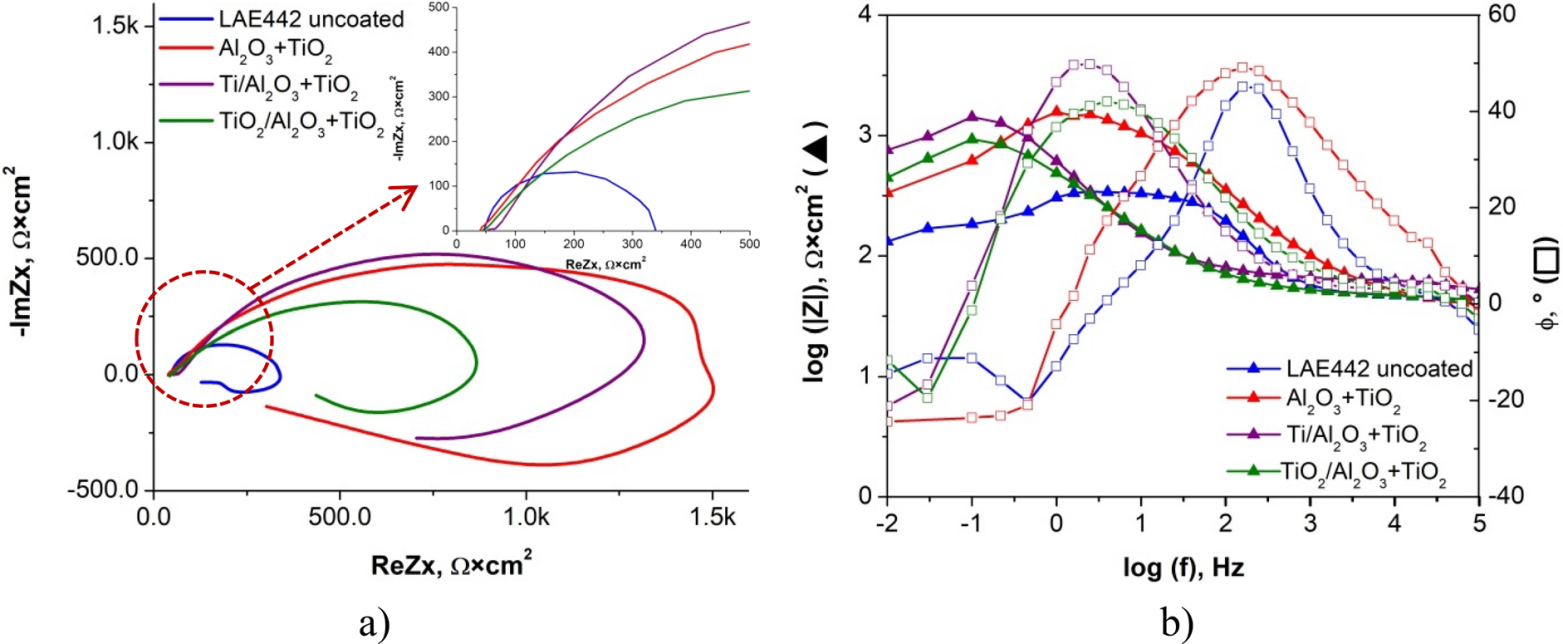
Results of electrochemical impedance spectroscopy (EIS) for uncoated and coated LAE442 alloy substrate: (**a**) the Nyquist representation, (**b**) the Bode representation.

**Figure 16:**
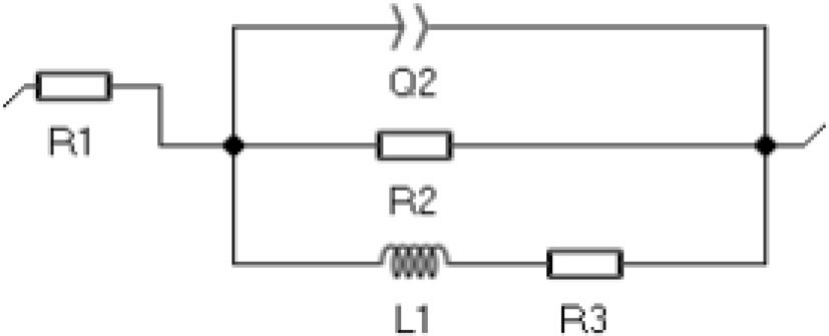
An equivalent circuit that represents the impedance spectra.

To more fully characterise the electrochemical properties of the produced materials with coatings, impedance spectroscopy tests were carried out for them, consisting in recording impedance spectra in the frequency range from 100 kHz to 10 MHz. The recorded results are shown in the Nyquist and Bode plots for the substrate material AE42 in Fig. 14. and the LAE442 alloy in Fig. 15. For the curve samples obtained during the test, the equivalent electrical circuit that best describes the corrosion system was fitted (Fig. 16), which consists of 4 elements, including a constant-phase CPE, resistors and an inductor, and its resultant impedance can be written as the following Eq. (1). Table 4 shows the calculation results matching the experimental ones, which are the parameters of the elements of the adopted equivalent circuit.

**Table 4:** Electrochemical impedance spectroscopy parameters.

Substr	Coating	R1, Ω	Q2, µF × s^(α-1)^	α2	R2, kΩ	L1, kH × cm^-2^	R3, kΩ
**AE 42**	Uncoated	41.11	9.5	0.93	1 72	355.6	1 932
	Al_2_O_3_ + TiO_2_	42.5	0.06	0.73	450	125	250
	Ti/Al_2_O_3_ + TiO_2_	42.9	2.2	0.77	5.6	3.7	1.6
	Ti + TiO_2_/Al_2_O_3_ + TiO_2_	48.42	0.28	0.95	9.3	2.0	0.7
**LAE 442**	Uncoated	45.44	13.4	0.92	0.3	0.1	0.2
	Al_2_O_3_ + TiO_2_	40.6	31.4	0.69	1.8	4.2	0.5
	Ti/Al_2_O_3_ + TiO_2_	52.85	224	0.92	3.2	1.0	1.7
	Ti + TiO_2_/Al_2_O_3_ + TiO_2_	43	50	0.95	0.9	0.6	0.8

For both coated magnesium alloys, the test results in the form of Nyquist curves show semi-circles with return segments in the low-frequency range. The slope angle of the curves (Fig. 14a) for the AE42 alloy with the coatings is clearly more significant than for the sample of the base material; the highest value was for the sample with the Al_2_O_3_ + TiO_2_ coating, for which also the recorded circle has the largest radius. The second, in terms of the quality of the corrosion protection, was the Ti + TiO_2_/Al_2_O_3_ + TiO_2_ coating, and then the sample with the Ti/Al_2_O_3_/TiO_2_ coating, which can be seen in the enlarged area of the Nyquist graphs.

The changes in impedance are shown in the form of a Bode diagram (Fig. 14b) allow the behaviour of the corrosion system to be tracked over a wide frequency range. The lowest impedance value in the entire range of tested frequencies was found for the base material sample, while the highest impedance value was recorded for the sample with the Al_2_O_3_ + TiO_2_ coating. The samples with the two remaining coatings, Ti/Al_2_O_3_ + TiO_2_ and Ti + TiO_2_/Al_2_O_3_ + TiO_2,_ had very similar impedance values, while the second of the mentioned materials ranged from medium to low frequencies was characterised by a higher impedance value. When analysing the second type of Bode plot (Fig. 14b), showing the dependence of the phase shift angle on the impedance modulus, it was clearly marked that the highest value of about 80° was recorded for the sample with the Al_2_O_3_ + TiO_2_ coating, a similar value of the phase shift angle was shown for the sample with the Ti + coating Ti + TiO_2_/Al_2_O_3_ + TiO_2_ but in half the frequency range. In contrast, the lowest result was recorded for the substrate material.

Based on the results of the electrochemical impedance spectroscopy method for the LAE442 alloy with various types of coatings, it can be concluded that their use improved the anticorrosive properties of the material, as evidenced by the course of the Nyquist curves (Fig. 15a), based on what bigger ranges of circles were observed for all coatings (higher value radius). The highest value of the curve slope was recorded for the Al_2_O_3_ + TiO_2_ and Ti/Al_2_O_3_ + TiO_2_ coatings, almost identical throughout the test, similar behaviour, but only in the high-frequency range, was characteristic for the sample with the Ti + TiO_2_/Al_2_O_3_ + TiO_2_ coating.

Analysing the course of Bode plots (Fig. 15b) for the LAE442 alloy, it can be concluded that the highest impedance value in the broadest range of tested frequencies was found for the sample with Al_2_O_3_ + TiO_2_ coating, only in the high-frequency range, the higher impedance value was recorded for the Ti/Al_2_O_3_ + TiO_2_ coating.

Based on the Bode diagram (Fig. 15b), showing the dependence of the phase shift angle on the impedance modulus, it was clearly marked that the highest value of about 50° was recorded for the sample with the Al_2_O_3_ + TiO_2_ coating in the range between medium and low-frequency values and for the Ti/Al_2_O_3_ coating + TiO_2_ in the range between high and medium frequency values. For each coated material, there was a wider range with increased angle values than for the substrate material, whose maximum value of the shift angle was 45° and in a very narrow range of the tested frequencies.

Comparing the results of the EIS tests on the AE42 and LAE442 alloys, it can be concluded that in the case of the first one, better results for the improvement of the characteristic electrochemical values were obtained: higher values of impedance and higher values of the phase shift angle is wider frequency ranges.

1 $$Z=R1+\frac{R2(j2\pi fL1+R3)}{j2\pi fL1\left(1+{\left(j2\pi f\right)}^{\alpha 2}Q2R2\right)+R3+R2(1+{\left(j2\pi f\right)}^{\alpha 2}Q2R3)}$$

The results of the measurements of the surface contact angles of the tested materials and the determined surface free energy (SFE) are summarised in Table 5. As a result of the contact angle measurement with a drop of water, it was found that both uncoated substrates and all tested coatings on the substrate made of magnesium and lithium LAE442 had hydrophilic properties. The contact angles θ for these surfaces range from 23° to 84°. The remaining samples, i.e. the tested coatings on a lint-free substrate, show hydrophobic properties as the contact angle θ is within the range of 99° ÷ 103°. The values of the diiodomethane contact angles for all tested samples are in the range of 53° ÷ 69°, and in most cases, the higher values of the θ angles are for coatings on a lithium substrate than for the same type of coatings on a lithium-free substrate. The values of the surface free energy depend on the type of substrate. The uncoated AE42 alloy and the SFE covered with the tested coatings range from 31 to 39 mJ/m^2^. The free surface energy for the LAE442 samples uncoated and covered with the tested coatings range from 51 to 95 mJ/m^2^. The SFE component values also show significant differences depending on the type of substrate. For lithium-free samples, much higher values are assumed for non-polar components, which proves that these materials show a greater affinity for the dispersion groups of SFE. In the case of samples of uncoated and coated magnesium alloys with lithium, polar components take higher values, so these materials have a greater affinity for polar SFE groups.

**Table 5:** The contact angle measurements and the surface energy calculated by the Owens–Wendt method.

Substrate	Coating	Wetting angle θ, °		Surface free energy, mJ/m^2^		
		Distilled water	Diiodomethane	γ_p_^S^	γ_d_^S^	γ_S_
**AE42**	Uncoated	84 ± 7.4	56 ± 0.4	6	25	31
	Al_2_O_3_ + TiO_2_	99 ± 2.0	58 ± 0.4	1	30	31
	Ti/Al_2_O_3_ + TiO_2_	103 ± 1.8	57 ± 0.7	0	35	35
	Ti + TiO_2_/Al_2_O_3_ + TiO_2_	102 ± 1.4	60 ± 0.5	0.1	32	39
**LAE442**	Uncoated	48 ± 2.6	53 ± 0.7	36.99	13.99	51
	Al_2_O_3_ + TiO_2_	38 ± 7.3	69 ± 0.6	85.01	30.03	95
	Ti/Al_2_O_3_ + TiO_2_	41 ± 6.4	69 ± 2.1	54.96	5.52	60
	Ti + TiO_2_/Al_2_O_3_ + TiO_2_	23 ± 5.8	61 ± 1.9	66.38	6.38	73

**Figure 17:**
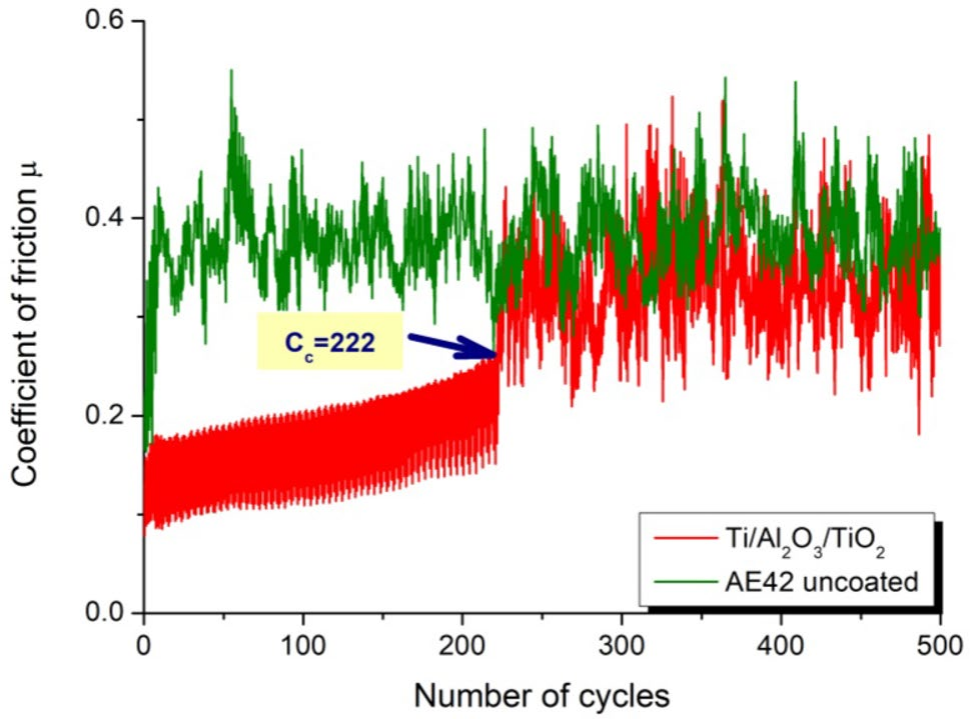
Friction coefficient as a function of the number of cycles for uncoated AE42 alloy substrate material and Ti/Al_2_O_3_ + TiO_2_ coating obtained by the hybrid method.

**Figure 18:**
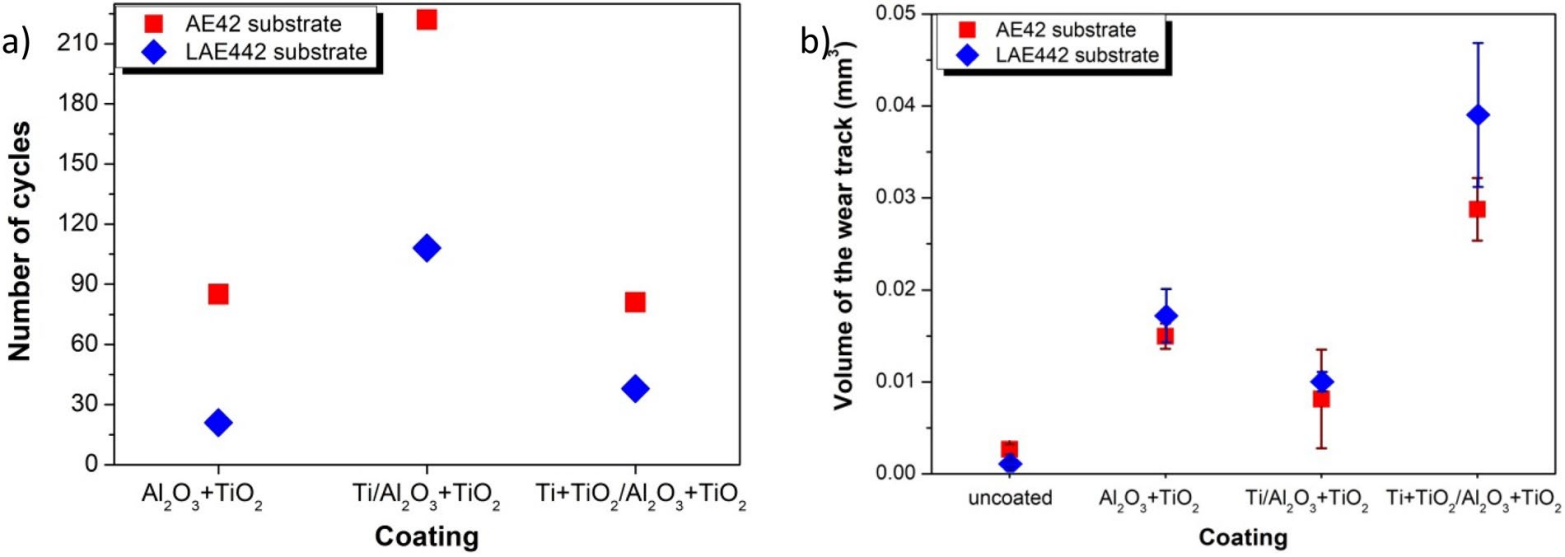
Comparison of the results of tribological tests of the investigated coatings on magnesium alloys: (**a**) the critical number of cycles, (**b**) the volume of the wear track.

As a result of the tests of abrasion resistance using the Ball-on-plate method, the critical number of C_c_ cycles was determined for all tested coatings. The value of this coefficient determines after how many friction cycles, under the assumed test conditions, the coating breaks and the substrate is exposed. In the first stage, the uncoated substrates were examined, and the friction coefficient for uncoated substrates was determined, which in both cases was about µ ≈ 0.4. This value is close to the values in the literature for magnesium alloys. The coated samples were then tested. The value of the friction coefficient of the samples covered with the tested coatings is lower and is in the range of µ ≈ 0.15 ÷ 0.2 (Fig. 17). After reaching a critical number of C_c_ cycles, the friction coefficient increases rapidly to a value close to the value of the substrate friction coefficient (i.e. µ ≈ 0.4). At this test stage, the coating is rubbed, and the substrate is exposed. As a result of the study, it was found that the Ti/Al_2_O_3_ + TiO_2_ coating has the highest value of the critical number of cycles, for which the C_c_ value is 222 and 108 cycles, respectively, for the coated AE42 and LAE442 substrates (Fig. 18a). Moreover, it should be noted that each type of coating exhibits a higher C_c_ value, and thus a higher abrasion resistance, on lithium-free substrates than on magnesium-lithium alloys.

Moreover, based on the measurements of the cross profiles of the wear track, the volumes of the rubbed material, i.e. the volumetric wear, were determined (Fig. 18b). As a result of the study, it was found that the lowest abrasion volume is characteristic for uncoated substrates, for which the average abrasion volumes are 0.003 mm^3^ and 0.001 mm^3^ for AE42 and LAE442 alloys, respectively. Among the tested coatings, the lowest volumetric wear is shown by the Ti/Al_2_O_3_ + TiO_2_ coating on both substrates, for which the volumes of the used material are 0.008 mm^3^ and 0.011 mm^3^ for coatings on magnesium alloys, successively with lithium and with lithium. The highest wear by volume is characteristic for materials with TiO_2_/Al_2_O_3_ + TiO_2_ coating, for which the volumetric wear is 0.029 mm^3^ and 0.041 mm^3^ for coated alloys AE42 and LAE442, respectively.

**Figure 19:**
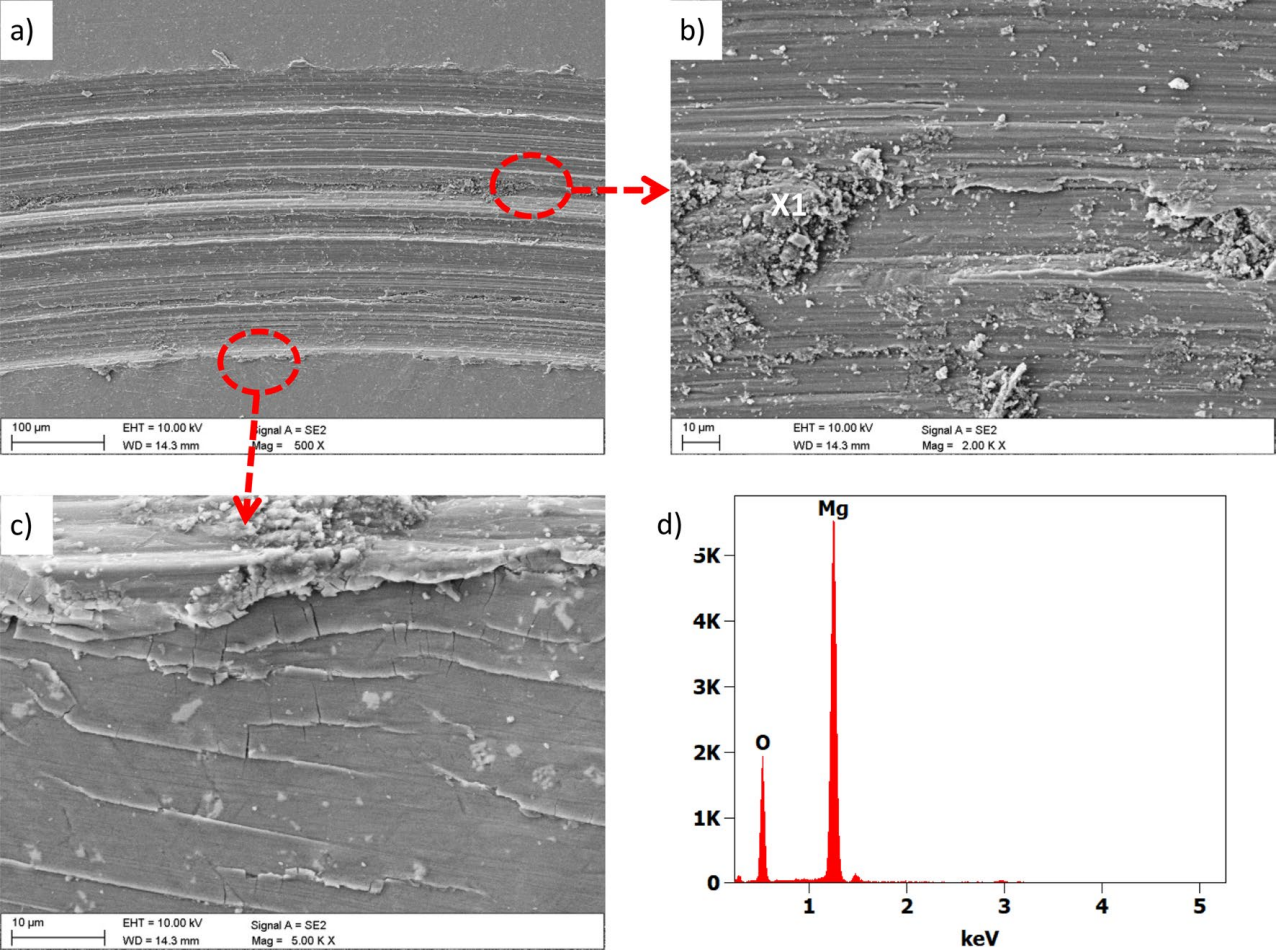
Wear trace after the "ball-on-plate" wear test for the Ti + TiO_2_/Al_2_O_3_ + TiO_2_ hybrid coating on AE42 alloy substrate, (**a**–**c**) SEM images, (**d**) X-ray energy dispersive plot of the area X1 shown in (**b**).

**Figure 20:**
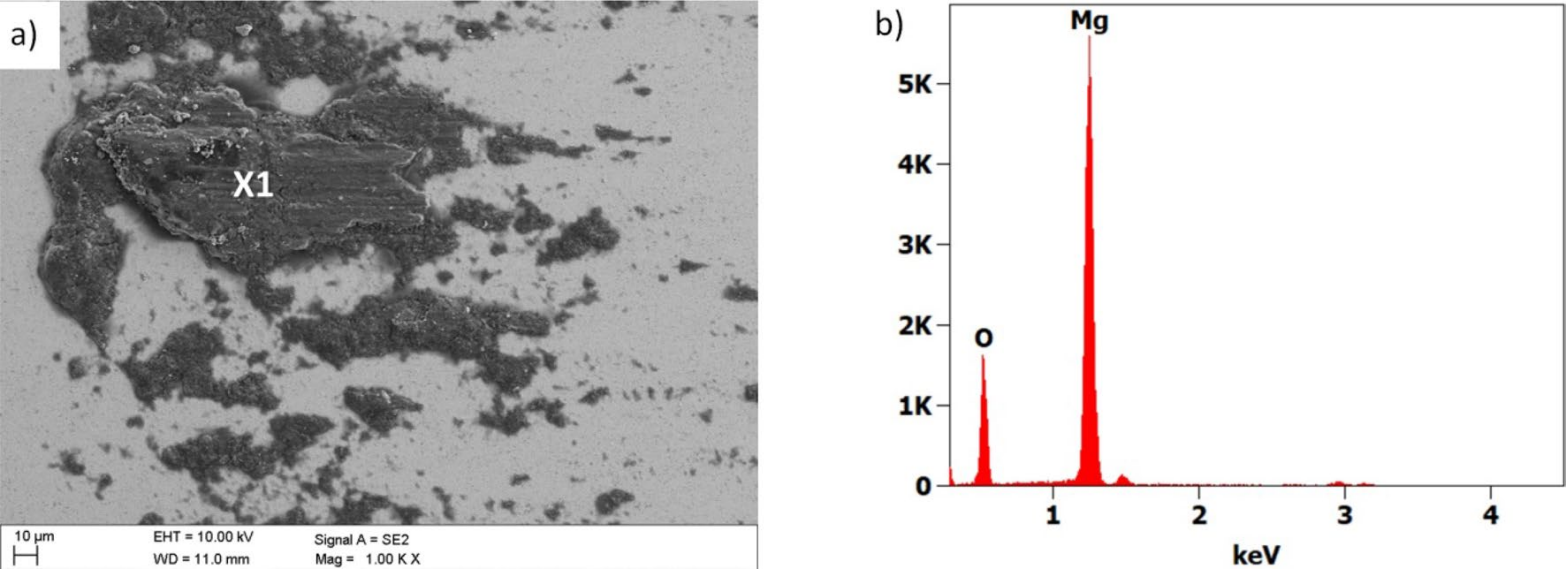
(**a**) Wear place after the "ball-on-plate" wear test for the cemented carbides ball as a counter-sample, (**b**) X-ray energy dispersive plot of the area X1 shown in figure (**a**).

Based on the observation of the abrasion paths in the SEM microscope, it was found that the primary wear mechanism was abrasion (Fig. 19). Abrasion completely removes the coating and exposes the substrate. In addition, tensile damage to the coating was found at the periphery of the tracks. There are numerous zones of magnesium oxides within the abrasion path, as evidenced by the EDS analysis (Fig. 19d). The study of the counter-sample made of cemented carbides showed that small layers of oxidised magnesium accretion were formed on its surface (Fig. 20). Other damage, such as abrasion on the counter-sample, practically does not occur.

## Summary

In many industry fields, the application of magnesium-lithium alloys is limited by their undesirable properties, mainly poor resistance to corrosion. The subject of these alloys has recently been strongly accented in the literature. If the mechanical properties of these alloys can be effectively improved by adding alloying components and precipitation hardening and cold forming, their corrosion resistance remains an unresolved problem. The paper presents the results of tests of hybrid coatings produced by methods combining the technologies of PVD and ALD, such as Ti/Al_2_O_3_ + TiO_2_ and Ti + TiO_2_/Al_2_O_3_ + TiO_2_, and ALD coatings, such as Al_2_O_3_ + TiO_2_. The magnesium alloys AE42 (Mg–4Al–2RE) and LAE442 (Mg–4Li–4Al–2RE) are the substrates used. As a result of structural tests in the transmission electron microscope, the layered structure of the coatings was confirmed. It was also found that the layers obtained by the PVD technique show a crystalline structure, and the titanium oxide of the ALD layers also contains nanocrystalline precipitates in an amorphous matrix.

Performed electrochemical investigations allowed us to evaluate the corrosion resistance of the materials studied. In particular, the corrosion current density decreased by 11 × for the sample with Ti + TiO_2_/Al_2_O_3_ + TiO_2_ coating relative to the substrate with AE42, and for the LAE442 alloy, the current density for this coating decreased by almost 14x. The improvement of the anti-corrosion properties of the applied coatings was also confirmed by the increased values of the polarisation resistance; in the case of the AE42 alloy, its value increased by 27% for the Ti + TiO_2_/Al_2_O_3_ + TiO_2_ coating, while for the LAE442 alloy and the same coating, the resistance value increased more than ten times.

Additional spectroscopic examination in the alternating current system and recording of the impedance spectra in the form of Nyquist and Bode diagrams allowed us to determine a more complete characteristic of the electrochemical properties of the produced coatings. The significantly higher value of the slope of the obtained curves for the coated materials, in the case of both tested alloys AE42 and LAE442, allows us to state that they were characterised by higher resistance in the tested environment of the aqueous NaCl solution and were subject to corrosion processes more slowly.

The significant improvement in corrosion resistance of the tested magnesium alloys covered with hybrid coatings should undoubtedly be explained by the mechanism of synergistic interaction of PVD and ALD layers. The dense ALD bilayer seals the crystalline PVD coating. In addition, the amorphous structure of the outer titanium oxide layer reduces charge transport and affects the reduction of corrosion current. The combination of PVD and ALD coatings makes obtaining electrochemical properties impossible to achieve with each technique separately. This is evidenced by the fact that in the case of coatings deposited in conventional (non-hybrid) processes, coatings consisting of titanium and titanium oxide layers by PVD on such substrates do not improve corrosion resistance TiO_2_ ALD coating shows an improvement in electrochemical properties. The results of tests of single PVD and ALD coatings are presented in^14^. It should be noted that the most significant benefit of the use of hybrid coatings is visible in highly reactive magnesium-lithium alloys.

**Figure 21:**
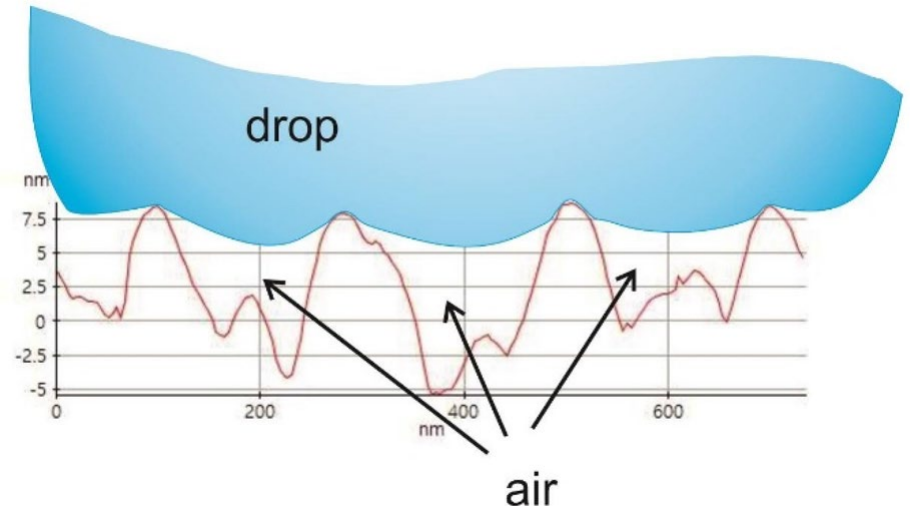
The nanoscale profile of the surface topography of the Ti + TiO_2_/Al_2_O_3_ + TiO_2_ coating on the AE42 alloy substrate obtained by the AFM analysis according to Fig. 1b together with the Cassie-Baxter wetting mechanism scheme.

The surface's wettability depends both on the chemical composition of the surface to be wetted and its morphology. Metal oxides are inherently hydrophilic; however, adsorbed carbon pollutants may contribute to the hydrophobicity of their surface^27–29^. As shown by the contact angle measurements, the lithium-free magnesium alloys covered with the tested coatings show hydrophobic properties, and both the coated and uncoated magnesium-lithium alloys are hydrophilic. In the tested materials, the wettability is strongly dependent on the morphological features of the coatings. The surfaces of nanoscale coatings on AE42 alloys show high roughness and a highly developed "coronal" morphology. Therefore, the mechanism responsible for the hydrophobicity of these surfaces is the Cassie-Baxter mechanism^30^. The hydrophobic state is due to the interphase of air bubbles trapped between the liquid and the solid (Fig. 21). On the other hand, the hydrophilicity of the coating surfaces on magnesium and lithium alloys can be explained by the action of the mechanism according to Wenzel's theory^31^.

Moreover, the XPS analysis confirmed the presence of carbon impurities on the surface, which also improves wettability. It must be highlighted that the morphology of the investigated coatings was dependent on the microstructure of the Al_2_O_3_ layer obtained by the ALD method. In the case of ruthless substrates, a layer. In the case of lithium-free substrates, the ALD layer consists of an aluminium oxide/titanium oxide bilayer. In the case of lithium-containing substrates, due to their diffusion from the substrate into the coating, a LiAlxOy layer is formed, which affects the different nature of the microstructural structure of the coating and its morphology. This, in turn, largely determines the contact angle and other properties, in particular electrochemical properties. The formation of lithium-aluminium oxide has already been presented in the works of Wang et al.^24,25^. The work^32^ presented a study in which a layer of pure crystalline aluminium was used as a sublayer under the aluminium oxide. This effectively blocked lithium diffusion from the substrate into the resulting Al_2_O_3_ layer. In contrast, the titanium and rutile-TiO_2_ sublayers used in this study did not provide a sufficient barrier to Li diffusion from the substrate into the coating.

Studies of tribological properties show that thin oxide coatings improve the tribological contact of the surfaces of the tested materials with the counter-specimen made of cemented carbides. It was found that the friction coefficient of the coated magnesium alloys was decreased concerning the uncoated materials. Moreover, it has been shown that for each tested coating, coatings on non-lite substrates are characterised by higher abrasion resistance than those on magnesium-lithium alloys. However, the observed increase in the volume of the worn material of coated samples concerning the uncoated substrate is because the torn oxide coatings, after exceeding the C_c_ value, constitute a micro-abrasive fraction in the further wear process, which intensifies this destructive process.

The recommended coating for applications on magnesium alloys, particularly ultra-light magnesium alloys with lithium, is the hybrid coating Ti + TiO_2_/Al_2_O_3_ + TiO_2_, which provides the best electrochemical properties among the tested coatings.

## Conclusions

Based on the research, the following conclusions were drawn:

- The Ti + TiO_2_/Al_2_O_3_ + TiO_2_ coating shows the best electrochemical properties in the potentiodynamic test among the tested coatings.
- The production of hybrid coatings of the Ti + TiO_2_/Al_2_O_3_ + TiO_2_ type allows for obtaining high electrochemical properties of the surfaces of the coated magnesium alloys of the AE42 and LAE442 type through the synergistic interaction of the combined layers obtained with PVD/ALD techniques, which is impossible to obtain with each of the techniques separately.
- The tested coatings on the AE42 substrate show hydrophobic properties, and on the LAE442 substrate hydrophilic properties, wettability is dependent on morphology, and in addition, the Cassie-Baxter and Wanzle mechanisms for coatings on AE42 and LAE442 substrates are the mechanisms responsible for wettability.
- The LiAl_*x*_O_*y*_ layer reappears by diffusion of lithium from the LAE442 substrate into the coating in place of the alumina. The diffusion of lithium takes place through a layer of rutile TiO_2_ obtained by PVD on the coated substrate.
- The tested coatings improve tribological contact by reducing the coefficient of friction in the friction node tested sample-ball WC–Co.

## Data Availability

All data generated or analysed during this study are included in this published article.
